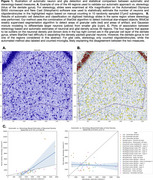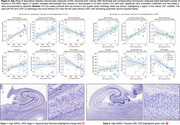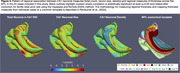# Associations between Quantitative Neuronal Loss Measures and Postmortem MRI Atrophy Measures in the Human Medial Temporal Lobe in AD and LATE

**DOI:** 10.1002/alz70856_103753

**Published:** 2025-12-26

**Authors:** Paul A. Yushkevich, Sadhana Ravikumar, Laura E.M. Wisse, Sydney A. Lim, Lisa M Levorse, Amanda E Denning, Ranjit Ittyerah, Petter Clemensson, Niyousha Sadeghpour, Karthik Prabhakaran, Winifred Trotman, Maria Mercedes Iniguez de Onzono Martin, Esther Buendia, Alicia Vela, Monica Munoz, Jose Carlos Delgado Gonzalez, Maria del Pilar Marcos Rabal, Sandra Cebada Sanchez, Carlos de la Rosa Prieto, Noemi Villaseca, Daniel T Ohm, John L. Robinson, Theresa Schuck, M. Dylan Tisdall, David J. Irwin, Eddie B Lee, Christopher D. Brown, Sandhitsu R. Das, David A. Wolk, Maria del Mar Arroyo Jimenez, Ricardo Insausti, Ana M Insausti

**Affiliations:** ^1^ Perelman School of Medicine, University of Pennsylvania, Philadelphia, PA, USA; ^2^ Penn Alzheimer's Disease Research Center, University of Pennsylvania, Philadelphia, PA, USA; ^3^ Kitware, Inc, Carrboro, NC, USA; ^4^ Department of Clinical Sciences Lund, Lund University, Lund, Lund, Sweden; ^5^ University of Pennsylvania, Philadelphia, PA, USA; ^6^ Kings College, London, London, United Kingdom; ^7^ University of Castilla‐La Mancha, Albacete, Spain; ^8^ University of Castilla La Mancha, Albacete, Castilla La Mancha, Spain; ^9^ Penn Frontotemporal Degeneration Center, Department of Neurology, Perelman School of Medicine, University of Pennsylvania, Philadelphia, PA, USA; ^10^ Department of Pathology and Laboratory Medicine, Institute on Aging and Center for Neurodegenerative Disease Research, The Perelman School of Medicine at the University of Pennsylvania, Philadelphia, PA, USA; ^11^ Center for Neurodegenerative Disease Research, Perelman School of Medicine, University of Pennsylvania, Philadelphia, PA, USA; ^12^ Penn Image Computing and Science Laboratory (PICSL), University of Pennsylvania, Philadelphia, PA, USA; ^13^ Department of Neurology, Perelman School of Medicine, University of Pennsylvania, Philadelphia, PA, USA; ^14^ Public University of Navarra, Pamplona, Navarra, Spain

## Abstract

**Background:**

Medial temporal lobe (MTL) atrophy measured on MRI is a sensitive biomarker of AD‐linked neurodegeneration but is not specific to AD. LATE is a common AD co‐pathology that is challenging to distinguish from AD based on available validated biomarkers. LATE and AD are both associated with hippocampus and entorhinal cortex atrophy, but recent studies suggest that there are differences in the severity and spatial patterns of atrophy. We sought to use postmortem MRI and histology to examine how MRI morphometric measures in AD and LATE relate to direct measures of neurodegeneration, including neuron number, size, and density.

**Method:**

Thionin‐stained 50µm histology sections from 24 brain donors (5 AD‐LATE‐, 14 AD+LATE‐, 5 AD+LATE+) with available 9.4T postmortem MRI were used to delineate subfields CA1, subiculum and entorhinal cortex. Deep learning method StarDist was used to detect star‐shaped objects in thionine slides, combined with weakly‐supervised learning to identify artifact‐free cortical regions and Gaussian mixture modeling to distinguish neurons from glia (Figure 1AB). Validation against stereology measures of neuronal/glial density was performed in 50 separate regions. Neuronal measures were compared to MTL volume and thickness measures extracted from MRI.

**Result:**

Automated neuronal density estimates (r=0.72, Figure 1C) agreed with stereology, but not glial density. Estimated number and size of neurons in CA1 and ERC were higher in individuals with less CA1/ERC atrophy (Figure 2) consistent with greater neuronal loss in advanced AD and LATE. However, CA1 neuronal and glial density, and ERC glial density, were higher in individuals with greater atrophy, suggesting tighter packing of neurons in remaining tissue and significant contribution of neuropil loss to MRI‐based atrophy measures. Pointwise analysis in Figure 3 shows patterns of association between regional MTL thickness and CA1 neuronal count, size, and density measures.

**Conclusion:**

These initial feasibility results in two sections in 24 brain donors encourage us to apply this pipeline to a larger dataset of paired postmortem MRI and *serial* histology sections (Ravikumar et al., 2024) and to study associations between tau pathology, neuronal loss, and MRI‐based measures of atrophy, with the aim of better differentiating atrophy linked to LATE and AD.